# Retinoic acid synthesis and functions in early embryonic development

**DOI:** 10.1186/2045-3701-2-11

**Published:** 2012-03-22

**Authors:** Richard Kin Ting Kam, Yi Deng, Yonglong Chen, Hui Zhao

**Affiliations:** 1 School of Biomedical Sciences, Faculty of Medicine, The Chinese University of Hong Kong, Shatin, New Territories, Hong Kong, P. R. China; 2 Department of Medicine and Therapeutics, Faculty of Medicine, The Chinese University of Hong Kong, Shatin, New Territories, Hong Kong, P. R. China; 3 Center for Molecular Medicine, Guangzhou Institute of Biomedicine and Health, Chinese Academy of Sciences, Guangzhou, P. R. China; 4 Key Laboratory for Regenerative Medicine, Ministry of Education, Ji Nan University-The Chinese University of Hong Kong, Shatin, New Territories, Hong Kong, P. R. China

**Keywords:** retinoids, retinoic acid synthesis, embryonic development, organogenesis

## Abstract

Retinoic acid (RA) is a morphogen derived from retinol (vitamin A) that plays important roles in cell growth, differentiation, and organogenesis. The production of RA from retinol requires two consecutive enzymatic reactions catalyzed by different sets of dehydrogenases. The retinol is first oxidized into retinal, which is then oxidized into RA. The RA interacts with retinoic acid receptor (RAR) and retinoic acid X receptor (RXR) which then regulate the target gene expression. In this review, we have discussed the metabolism of RA and the important components of RA signaling pathway, and highlighted current understanding of the functions of RA during early embryonic development.

## Introduction

Retinoids refer to those chemicals that are structurally or functionally similar to retinol, or vitamin A [1], which is an essential biomolecule for embryonic development and adult body homeostasis. All retinoids retain the polyene hydrophobic tail attached to a cyclic 6-carbon ring. The polyene tail is characterized by the alternating conjugated carbon-carbon double bonds, which makes retinoids light-sensitive. In contrast with other signaling proteins, retinoids have a much lower molecular weight of approximately 300 Da. Given their molecular structures, retinoids are highly oil-soluble and able to diffuse across the cell membrane. Retinoids are involved in cellular growth, apoptosis, immune response, and epithelial growth [2-7] through the interaction with the nuclear receptors, retinoic acid receptor (RAR) and retinoid X receptor (RXR). During early embryonic development, the major active form of retinoids, all*-trans *retinoic acid (atRA), regulates germ layer formation, body axis formation, neurogenesis, cardiogenesis, and the development of pancreas, lung, and eye. It is also a critical element for visual function [8]. Because of the wide spectrum of RA functions, the metabolism, regulation, and function of vitamin A have been extensively studied for decades, and here we summarize our current understanding on retinoids metabolic pathways and RA functions during early embryonic development.

## 2. Metabolism of vitamin A and the production of all-*trans *retinoic acid

Vitamin A is a necessary dietary vitamin for the normal development and vision. The critical necessity of vitamin A was hinted as early as 1881 by Nikolai Lunin, who discovered that purified protein, fat, and carbohydrate did not sustain the normal growth of mice, unless the diet was supplemented with milk. Elmer Verner McCollum, then determined in 1917 that the critical component concerned in milk was actually a "fat-soluble factor A", named in contrast to the previously discovered "water-soluble factor B", or vitamin B. These discoveries allowed Carl Edvard Bloch, a Denmark paediatrician, to identify vitamin A deficiency as the cause of night blindness, or xerophthalmia [9].

**Figure 1:**
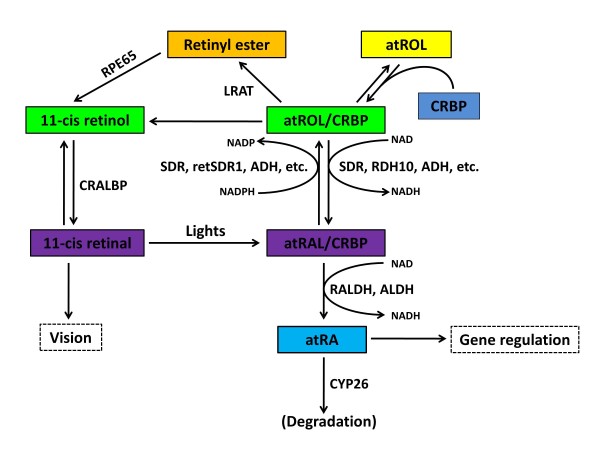
**Schematic diagram showing the metabolic pathways of vitamin A metabolism**. This illustration lists the major steps of RA metabolism in cell, please refer to the text for details. Abbreviation: NAD, nicotinamide adenine dinucleotide; NADH, The reduced form of NAD; NADP, nicotinamide adenine dinucleotide phosphate; NADPH, The reduced form of NADP; CRBP: cellular retinol-binding protein; LRAT: lecithin retinol acyltransferase; REH: retinyl ester hydrolase; ADH: alcohol dehydrogenase; RDH: retinol dehydrogenase; SDR: short-chain dehydrogenase/reductases; ALDH: aldehyde dehydrogenase; RALDH: retinaldehyde dehydrogenase. CRAD: *cis-*retinoid/androgen dehydrogenase. Modified from [18,25].

While vitamin A was a necessary dietary vitamin, vitamin A itself is not the main bioactive mediator of its function. The key mediators of vitamin A function were identified as atRA and 11-*cis *retinal. atRA is a regulator of gene transcription, while 11-*cis *retinal acts as a chromophore for visual functions [10]. In this section, we will review the metabolic processes of converting vitamin A into various retinoids, with emphasis on the production of atRA (Figure 1).

### 2.1 Conversion of Vitamin A (atROL) to all-trans retinal (atRAL)

Vitamin A (hereby referred to as all-*trans *retinol, atROL) is absorbed in the small intestine and esterified as retinyl esters for the blood stream transport. Retinyl ester is first transported to the liver for storage, mainly in the hepatic stellate cell. Hydrolysis of retinyl esters results in retinol, which then binds to retinol binding protein (RBP). The atROL/RBP complex is the dominant form for systematical and intercellular transport. After target organs take up the atROL/RBP complex, the atROL either is re-esterified into retinyl ester by lecithin retinol acyltransferase (LRAT) or binds to cellular retinol-binding protein (CRBP). The CRBP can prevent intracellular atROL from non-specific oxidation, immobilize intracellular retinol for storage, and acts as a carrier protein to present the atROL to respective retinol dehydrogenase for oxidation, or to LRAT for esterification [11]. Thus, the esterification by LRAT and/or the binding to CRBP represent the most upstream regulation of atROL availability, and therefore atRA metabolism, in the cell [12].

Retinyl ester and CRBP-bound retinol are the main storage forms of atROL. The retinyl ester can be hydrolyzed into 11-*cis *retinol by isomerohydrolases [13,14]. The cellular retinaldehyde binding protein (CRALBP) binds to 11-*cis *retinol with high affinity [15], which leads to the oxidation of 11-*cis *retinol to 11-*cis *retinal by 11-*cis *retinol dehydrogenase [16,17]. The 11-*cis *retinal is an essential component for vision. It binds to opsin to form rhodopsin, which can absorb lights within the visible spectrum. When the 11-*cis*-retinal absorbs a photon, it isomerizes from the 11-*cis *state to atRAL. The binding of atRAL to opsin is not stable and atRAL is rapidly released from opsin. Such molecule movements cause cell membrane change, and eventually lead to generation of the nerve impulse for vision (Figure 1).

atROL/CRBP complexes are the first substrate in the metabolic pathway which leads to the production of atRA. Using atROL/CRBP as substrate, retinol dehydrogenase (RDH), which belongs to short-chain dehydrogenase/reductase (SDR) family, catalyzes the oxidation of atROL to all-*trans *retinal (atRAL) [18]. This step is the rate limiting step in the production of atRA [19]. It has also been demonstrated that *in vitro *free atROL can be converted into atRAL by non-specific enzymes including alcohol dehydrogenase [20]. CRBP therefore provides a selection mechanism for specific RDH-mediated oxidation of atROL. Indeed, RDH has a much higher affinity towards atROL/CRBP complex than free atROL and the reaction depends on protein-protein interaction between RDH and CRBP [21]. It has been demonstrated that the microsomal RDH interacts with atROL/CRBP-I in the presence of co-factor, nicotinamide adenine dinucleotide phosphate **(**NADP) [22]. Moreover, atROL/CRBP is the preferred substrate for the retinol dehydrogenase 16 (RDH16. Abbreviated as RoDH-4 in the original article), but not for 3α-hydroxysteroid dehydrogenase (3α-HSD), a similar alcohol dehydrogenase which has an even higher affinity towards free atROL [23]. Thus, CRBP acts as a selection protein for RDH by increasing the substrate affinity for RDH, which in turn prevents non-specific oxidation of atROL by alcohol dehydrogenase.

The conversion of atROL to atRAL is, in fact, reversible. The direction - oxidation or reduction - favored by different members of SDR family depends on the substrate affinity, co-factor affinity, and the rate of reaction. In addition to CRBP that contributes to substrate affinity, another factor that contributes to substrate affinity is the intrinsic substrate binding site on the SDR family of enzymes. Retinal reductase 1 (RalR1), a human SDR, has been shown to be reactive towards both atROL and atRAL *in vitro*. However, RalR1 has a higher affinity and rate of reduction for atRAL than atROL, indicating that RalR1 is a retinal reductase, rather than retinol dehydrogenase, under physiological conditions [24]. Co-factor affinity of different SDR has also been demonstrated that the co-factor favored by SDR reflects the direction of reaction catalyzed by that particular SDR (reviewed by Pares) [25].

Apart from RalR1, a number of retinal reductases have been identified in the past decades. A human short-chained retinol reductase (retSDR1) identified in a neuroblastoma cell line has been shown to promote the formation of retinyl ester in the presence of exogenous atRAL [26]. A mouse liver peroxisomal SDR termed mouse retinal reductase (RRD) also showed a high atRAL-specific reductase activity in the presence of CRBP *in vitro *[27]. This enzyme was induced by peroxisome proliferator-activated receptor (PPAR), suggesting a relationship between retinoid metabolism and peroxisome activity [27]. Using *in vitro *assay, some studies have identified reductases which showed *in vitro *retinal-reducing activity. For example, human aldose reductase and human small intestine aldose reductase can function as retinal reductase *in vitro *[28]. Similarly, the mouse short-chained aldehyde reductase (SCALD) could reduce atRAL and 9-*cis *retinal *in vitro *[29]. However, these studies are based on *in vitro *biochemical analysis only and do not take into account the substrate selection by CRBP. Therefore, the enzymatic activities on different retinal reported should not be taken as a direct evidence for physiological retinal reductases.

### 2.2 Conversion of atRAL to atRA

Similar to atROL, all-*trans *retinal is also transported by CRBP in the cell, and is then oxidized to atRA. The oxidation from atRAL to atRA has been observed as early as 1960 [30]. The oxidation of atRAL to atRA was mediated by various retinaldehyde dehydrogenases (RALDH). At least 3 RALDHs have been identified in human, mouse, and *Xenopus*, with different physiological functions [31]. Retinaldehyde dehydrogenase 1 (Raldh1) is highly expressed in the dorsal retina of mouse embryos [32], in epithelial tissues of adult mice and *Xenopus *[33], and in the stomach and small intestine of adult rats [34]. *Xenopus *raldh1 has been shown to be an atRA synthesizing enzyme in a retinoic acid responsive cell line [35]. *RALDH1^-/-^*mice also suggest that Raldh1 is capable of atRA synthesis [36]. However, knockout of *RALDH1 *did not severely affect the morphology of the retina although RALDH1 is localized in the dorsal retina [36], indicating that other enzymes might redundantly share the function of RALDH1.

The retinaldehyde dehydrogenase 2 (RALDH2) was identified in human, mouse, chick, zebrafish and *Xenopus *[37-39]. Interestingly, RALDH2 was identified as a crucial enzyme for atRA synthesis in different organisms. Knockout of *RALDH2 *was embryonic lethal during the post-implantation period in mice [40], suggesting that atRA is essential for normal embryonic development. The phenotypes of RALDH2 knock-out mice include severely impaired segmentation of rhombomeres, altered homeobox gene expression pattern, and defective neural crest cell migration [41]. In zebrafish, knockdown of *raldh2 *caused a down-regulation of retinoic acid signaling, malformation in the central nervous system, and disruption of left-right asymmetry [42,43]. The *raldh2 *mutant, neckless (nls), displayed a suppressed formation of the midbrain to hindbrain region, as well as segmentation defects in rhombomeres [44]. Such defects were attributed to the reduction in atRA signaling [45], since the spatial and temporal pattern of atRA signaling is maintained mainly by raldh2 and a degradative enzyme cytochrome P450 hydroxylase A1 (cyp26a1) in zebrafish. In *Xenopus *embryos, ectopic expression of *raldh2 *caused teratogenic effects such as the expression of posterior neural markers (*en2 *and *krox20*) in the anterior region, which is similar to that due to atRA toxicity [38], suggesting that raldh2 is an important enzyme in maintaining atRA homeostasis in embryos. Knockdown of *raldh2 *in *Xenopus *embryos caused a shortening of anteroposterior axis and a posterior shift of neural marker *en2 *and *krox20 *[46]. Collectively, such evidence indicated that *raldh2 *plays a crucial role in the anteroposterior patterning of the central nervous system and trunk axis through regulation of the RA signaling.

Retinaldehyde dehydrogenase 3 (RALDH3) has been identified in human, chick, mouse, zebrafish, and *Xenopus*, and is expressed in the ventral retina across various species [47-51]. Studies in mouse have shown that RALDH3 was mainly involved in the frontonasal development and patterning of ocular structures [52]. Mice lacking *RALDH3 *were neonatal lethal, due to the respiratory tract obstruction in nasal region, and the neonatal lethality could be rescued by atRA supplements [53], suggesting that RALDH3 is an atRA synthesis enzyme. In 2007, Halilagic *et al. *showed that atRA production by RALDH3 contributed to the correct patterning of the anterior and dorsal boundaries of the developing forebrain [54]. It was further delineated that RALDH3 knockout mice exhibited loss of dopamine receptor D2 in the ventral forebrain. These studies suggest that RALDH3 is essential for the development of the central nervous system and the morphogenesis of anterior head structures [52].

**Figure 2:**
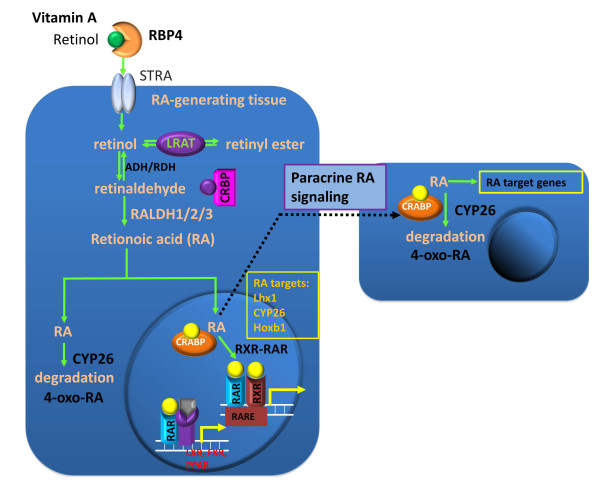
**Ilustration of RA and paracrine RA signaling**. In serum, retinol is bound to retinol-binding protein 4 (RBP-4) synthesized in the liver. Although retinol is lipid soluble, it enters cells mainly through the interaction with its receptor STRA. In the cell, retinol can either be converted into retinyl esters for storage via lecithin retinol acyltransferase (LRAT) or bind to the cellular retinol binding protein (CRBP). The CRBP-bound retinol is oxidized to retinal by either alcohol dehydrogenase (ADH) or retinol dehydrogenase (RDH), and retinal is oxidized to retinoic acid (RA) by retinaldehyde dehydrogenases (RALDH1/2/3). All-*trans *retinoic acid (atRA) is the major bioactive component among the retinoids. CYP26 can further oxidize atRA to 4-*oxo*-RA for degradation. Cellular retinoic acid-binding protein (CRABP) facilitates transportation of atRA into the nucleus where atRA binds its receptors. The ternary complex of ligand-bound RAR and RXR binds to the retinoic acid response element (RARE) and activates the RA target genes. atRA can diffuse to adjacent cells to activate target gene expression in these cells. RAR can also bind to the liver X receptor (LXR), farnesoid X receptor (FXR), and peroxisome proliferator-activated receptor (PPAR) for multiple functions.

Similar to atROL and atRAL, the metabolism of atRA is also closely related to retinoid binding protein termed cellular retinoic acid binding proteins (CRABPs). CRABPs bind to intracellular RA and prevent it from non-specific degradation [55,56]. There are two species of CRABP, CRABP-I and CRABP-II. These carrier proteins also ensure the solubility of hydrophobic retinoid in the aqueous intracellular environment. However, a recent study of CRBP-I/CRABP-I/CRABP-II triple knock-out mice has shown that the main regulator of retinoid homeostasis was likely to be CRBPs, with CRABPs playing a minor role in this process. Hoegberg *et al. *found that the chemical-induced depletion of total retinoids in triple knockout mice was more severe than the wild type and CRABP-I/CRABP-II double knockout mice [57], suggesting that CRBP-I is a more potent regulator of retinoid homeostasis. While CRABPs might not be critical in regulating total retinoids homeostasis, they participate in mediating RA signaling by transporting RA to the nucleus to interact with RARs. CRABP-II was shown to be translocated into nucleus upon the ligand binding [58], which allows atRA to bind to and activate RAR, a transcription factor responsible for the RA signaling (Figure 2). Interestingly, the RA signaling is tightly regulated by negative feedback mechanisms as CRABP-II is negatively regulated by atRA [59]. Elevated RA signaling suppresses the production of CRABPs, which down-regulate the activation of RARs and the RA signaling. CRABP-I, on the other hand, regulates the rate of RA metabolism by presenting RA to the degrading enzyme CYP26A1 [60].

### 2.3 Degradation of atRA

All-trans retinoic acid is degraded by CYP26 enzymes, which belong to cytochrome P450 hydroxylase family. A number of CYP26 family including CYP26A1, B1, C1, and D1 have been characterized and all of them possess the ability to degrade atRA into less bio-active retinoid [61-63]. Rhombomeric alteration defects were only observed by the knockdown of all three cyp26 enzymes in zebrafish [64], suggesting that cyp26a1, b1 and c1 act redundantly in hindbrain patterning. CYP26A1 is induced by atRA while it promotes the hydroxylation of atRA into 4-hydroxy retinoic acid, 4-oxo retinoic acid, and 18-hyroxy retinoic acid [45,65-67]. Since RALDH2 and CYP26A1 are both regulated by atRA itself, the metabolism of atRA therefore forms an auto-regulatory loop that regulates and balances atRA levels in embryos. Such regulation not only maintains the endogenous atRA level within a normal range, but also allows the organisms to respond to exogenous atRA fluctuation.

## 3. Retinoic acid receptors

atRA is carried into the nucleus by CRABP-II, and interacts with RARs, which themselves are transcription factors. RARs belong to retinoid receptor family, which also includes another group called retinoid X receptors (RXRs). RARs recognize both atRA and 9-*cis *retinoic acid, while RXRs only recognize 9-*cis *retinoic acid. Upon the ligand binding, RAR dimerizes with RXR to form a heterodimer, which then initiate gene transcription by binding to the retinoic acid response element (RARE) in the promoter region of the targets genes (Figure 2). The RAR family consists of RARα/β/γ three members that bind to atRA [68-71]. Single knockout mice that lack each of RARs were not embryonic lethal and did not display the complete spectrum of vitamin A deficiency phenotype. A disruption in RARα did not cause any observable phenotypic change in a mouse model [72]. Knockout of RARβ caused a reduction in the body weight and ocular defect, while limb formation remained normal [73]. Double knockout of two RARγ subtypes caused growth deficiency, cartilage dysmorphogenesis, and vertebrate malformation [74]. These results imply that RARs work redundantly and compensate the function of each other. Indeed, knockdown of RARα caused an increase in the expression level of RARβ and RARγ [75]. Only double knockout mutants showed phenotype close to the symptoms of vitamin A deficiency [76]. The auto-regulatory loop of *RAR *expression is similar to that regulating the expression of of *RALDH2 *and *CYP26a1*. Moreover, RARE has been identified in the promoter regions of *RARα *and *β *[77-79], indicating that the expression of these *RARs *is also under control of atRA.

Similarly, there are also three subtypes of RXRs [80]. RXRα knockout mice were embryonic lethal, potentially due to malformation of the heart in utero [81]. RXRβ knockout mice were 50% embryonic lethal, and the surviving littermates were morphologically normal except spermatogenesis defects which rendered the male sterile [82], while the RXRγ-null mutant mice were morphologically normal when compared with the wild type [83]. Moreover, the mice carrying only one copy of RXRα (RXRα+/-/RXRβ-/-/RXRγ-/-) were viable, suggesting one copy of RXRα is sufficient to carry out most of function of the RXRs [83]. Since atRA-bound RAR can form heterodimer with RXR in the absence of 9-*cis *retinoic acid and is still active in transcription activities, the importance of RXRs may not be as critical as RARs. This may explain why only one copy of RXRα is sufficient for the mouse embryonic development. Taken together, these results suggest that each of the RAR subtypes function redundantly and most of the RXR subtypes are not critical for the embryonic development.

While RARs mainly mediate the RA signaling, it has been revealed by many studies that ligand-bound RXRs activate other signaling pathways by forming heterodimer with other nuclear receptors such as liver X receptor (LXR), farnesoid X receptor (FXR), and PPAR [84-86] (Figure 2). LXR mainly functions as a sensor of cholesterol levels by recognizing its ligand oxysterols. Overloading cells with cholesterol activates LXR/RXR heterodimers which in turn initiate transcription of target genes, thereby regulate cholesterol transport, uptake, metabolism, and bile acid synthesis in the liver [87,88]. FXR can recognize free or conjugated bile acid and thus acts as an intracellular sensor of bile acid to regulate the metabolism of bile acid in the liver. Activation of liganded-FXR/RXR promotes bile acid efflux and inhibits bile acid synthesis [89]. PPAR is a lipid sensing nuclear receptor, recognizing a wide range of fatty acids [90]. These interactions between LXR, FXR, PPAR, and RXR reflect the complexity of RXR functions and their potentials on the RA signaling. In addition, not only do RXRs take part promiscuously in multiple signaling pathways, the expression of these nuclear receptors is also under control of complex feedback loops and cross-talks with other signaling pathways [91]. The heterodimerization of RXR with RAR, LXR, FXR, or PPAR is therefore mutually competitive, and atRA signaling not only triggers the transcription of its target genes, but also competitively suppresses the transcription of others. This may explain the board spectrum of atRA-induced teratogenicity observed in embryos.

## 4. Differential expression and gene regulation of RA metabolic enzymes

**Figure 3:**
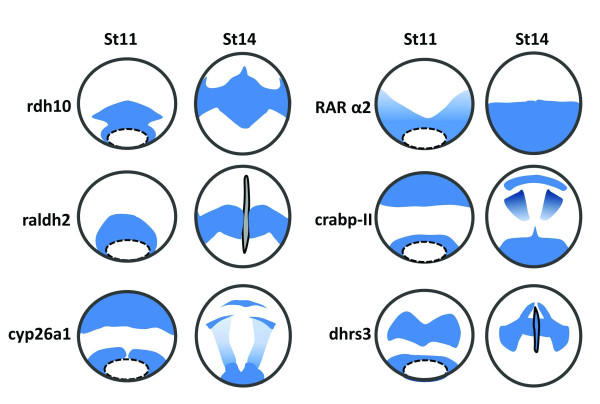
**Schematic diagram illustrating expression of the genes that are involved in RA biosynthesis and transportation at gastrula (stage 11) and neurula (stage 14) stages of *Xenopus *embryos**. *rdh10 *is expressed in the circumblastoporal region of *Xenopus *gastrula, and the signals at the dorsal side form a zone extending anteriorly. At stage14, the signals are found in trunk paraxial mesoderm region [46]. *rdh10 *is not expressed in the notochord. *raldh2 *is expressed in a form of a ring around the vegetal pole with signals more intense in the dorsal blastpore lip. During neurula stages, *raldh2 *signals are mainly distributed in the trunk paraxial mesoderm, expanding ventrally [38]. *rdh10 *and *raldh2 *display overlapping expression pattern in the trunk paraxial mesoderm, with *rdh10 *expressed more anteriorly than *raldh2. cyp26a *is expressed in two primary domains at stage11, the posterior domain surrounding the blastopore and the anterior domain covering the anterior part of prospective neural plate. At stage 14, the anterior *cyp26a *transcripts is developed into three elements corresponding to the cement gland anlage, the mid-/hindbrain boundary, and the auditory placodes, while the posterior expression domain remains in the circumblastoporal area, and the developing neural plate is also covered by a gradient of *cyp26a *signals with the highest present in posterior region [92]. The expression domains of *radlh2 *and c*yp26a *do not overlap at gastrula and neurula stages of *Xenopus *embryos. *rarα2 *is expressed in the involuting surface layer surrounding the blastopore, and becomes stronger as the gastrulation proceeds. At stage 14, *rarα2 *expression is expressed predominantly in the posterior neural plate of the embryos [167]. The expression of the *carbp-II *is defined into an anterior and a posterior domain at gastrula stages. In the anterior domain, *Xenopus crabp-II *is limited to the dorsal area which generates prospective head structures. At the neurula stages, *crabp-II *is expressed in the prospective telencephalon and rhombencephalon, and the most posterior region of the embryos [168]. The *dhrs3 *signals form a circumblastoporal ring which is similar to *rdh10 *at stage 11. The signals in the neural plate form two signaling zones and gradually converge towards the midline, forming two signal strips extending posteriorly. In addition, *dhrs3 *is expressed in the notochord at neurula stages [169]. All these drawings are shown in dorsal view and the blue color represents expression signals.

**Figure 4:**
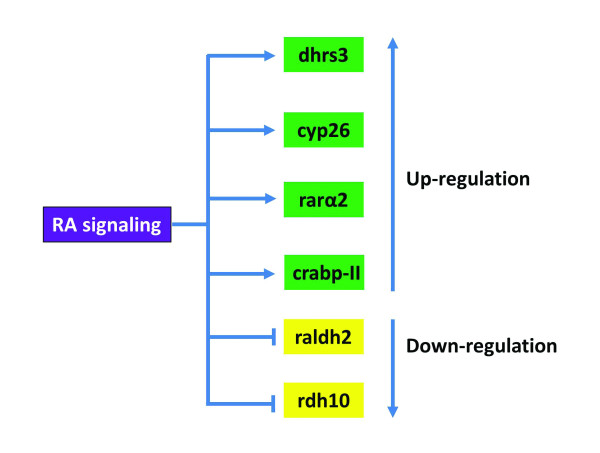
**The RA signaling regulates expression of the components involved in RA synthesis and transportation**.

The RA metabolic enzymes show distinct differential expression pattern during early embryonic development, and interestingly their expression is regulated by the RA signaling. Detailed descriptions of the expression patterns of these genes are beyond the scope of this review. Schematic drawing of expression of *rdh10, dhrs3, raldh2, cyp26*a1, *rarα2*, and *crabp-II *at *Xenopus *gastrula (stage 11) and neurula (stage 14) stages are illustrated in Figure 3, which shows that the RA signaling itself regulates expression of the enzymes for RA biosynthesis and elicits the complexity of RA acting as a morphogen in early embryonic development. Ectopic *cyp26 *expression can be induced by atRA treatment [92], while the embryos treated with atRA showed down-regulation of *raldh2 *[38] and *rdh10 *[46]. *Dhrs3 *can also be induced by atRA treatment (RKT Kam, Y Chen, WY Chan and H Zhao. Dhrs3 attenuates the retinoic acid signaling and is required for early embryonic patterning. Submitted). Thus the RA signaling down-regulates the expression of the enzymes for atRA production, but up-regulates enzymes that can reduce atRA level in embryos. Other components in the RA signaling are also responsive to atRA treatment. For example, *crabp-II *was found to be an atRA-inducible gene [93], and was found to contain a RARE domain in its promoter region [94]. Similarly, *rarα2 *was found to be inducible by atRA treatment in leukemic cell lines [95], and in rat embryos as well [96]. We summarize the regulation of these genes by RA signaling in Figure 4.

## 5. Retinoic acid signaling during early embryonic development

The RA signaling pathway has been implicated in various developmental processes. During early embryonic development, retinoids act as an important morphogen across different species from invertebrate to metazoan including human [97,98]. It participates in regulating various biological processes, such as apoptosis and differentiation, and cell fate specification.

### 5.1 Axis formation

The RA signaling has been implicated in embryonic axis formation as well. Evidence shows that it interacts with Nodal signaling to regulate dorsoventral axis formation. The mice embryos lacking all three *Cyp26 *genes displayed secondary body axes due to expansion of the Nodal expression domain. In fact, the mouse Nodal gene contains an RARE in the intron 1 that is highly conserved among mammals [99]. Moreover, pre-gastrulation mouse embryos express *Cyp26*s but not *Raldh*s suggesting that maternal RA is decreased by embryonic Cyp26s for proper Nodal expression during embryonic patterning [99].

In addition to the dorsoventral axis formation, *cyp26 *is also important for restricting the expression of posterior genes during the anteroposterior patterning [100]. Experiments in zebrafish embryos indicate that reduction of atRA by ectopic *cyp26 *decreased the expression of posterior genes *meis3*. Consistent with this, knockdown of *cyp26 *led to the anterior expansion of *meis3*. Interestingly, the expression of *cyp26 *is suppressed by the FGF and Wnt signalings, which are involved in the specification of posterior trunk during the gastrulation. Thus, RA together with FGF and Wnt signalings forms a complex regulatory network to regulate the anteroposterior axis formation in gastrula embryos, the *cyp26 *being one of the cross-talk genes linking these signaling pathways [100].

The RALDHs and CYP26s enzymes also participate in the anteroposterior patterning of the central nervous system by maintaining a gradient of atRA along the axis [101]. Global application of atRA to mouse embryos during day 7 of gestation caused severe head deformations, such as exencephaly, microcephaly, and anencephaly, whereas exposure of embryos to atRA during day 8 of gestation led to severe caudal truncations reminiscent of the human caudal regression syndrome [102,103]. Similar phenomena were observed with *Xenopus *embryos as well [104-106].

Vertebrates display asymmetric placement of various internal organs including the heart, liver, spleen, and gut, and the asymmetric development of paired organs such as brain hemispheres and lungs. Treatment with RA antagonist in mouse led to randomization of heart looping and perturbed sideness of node [107]. Application of RA antagonist revealed that the RA signaling sequentially controlled visceral and heart laterality [108]. On the other hand, the somites obviously avoid the influence of signaling pathways that regulate the left-right asymmetry. The somites, derived from the trunk paraxial mesoderm and sequentially segmented along the anteroposterior axis, are formed as bilaterally paired units. However, the mice embryos that lack *Raldh2 *exhibited asymmetric somite formation [109], which was due to left-right desynchronization of the segmentation clock oscillations. Such defects were also observed in RA deficient chick [110] and zebrafish [43]. It is therefore postulated that the RA form a protection zone, protecting the somite from the left-right asymmetric signals to maintain the bilateral symmetry of somites columns.

### 5.2 Neural differentiation

atRA has been known for its ability to induce neural differentiation. During neural differentiation in early embryonic development, the pro-neural induction factor Neurogenin 2 (Neurog2) is required for primary sensory neuron specification [111]. Two RARE identified in the promoter region of mouse *Neurog2 *provide evidence that atRA directly regulates the expression of *Neurog2 *and therefore neural differentiation [112]. In addition, it has been shown that atRA treatment can induce pluripotent embryonic carcinoma stem cell line NT2 cells to differentiate into forebrain, hindbrain, and spinal cord neural progenitors [113], and the NT2 cells displayed GABAergic and glutamatergic phenotype. In line with this, mouse embryonic stem cells could be induced into GABAergic neurons by atRA treatment *in vitro *[114]. These *in vitro *findings were also supported by an *in vivo *study that knockdown of *RARα *abolished the effect of atRA on dendritic growth [115]. Collectively, these studies support the view that atRA is required for neural differentiation in the central nervous system.

### 5.3 Hindbrain patterning

**Figure 5:**
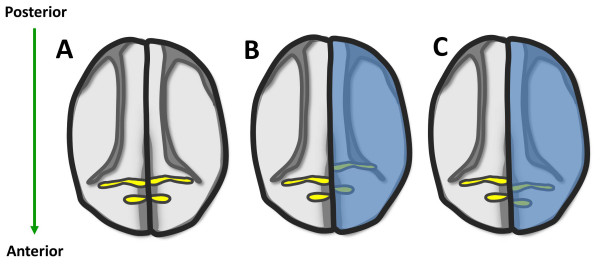
**Schematic diagram of *krox20 *expression in control embryos (A), and in embryos with alterated RA signaling (B, C)**. Alternation of RA gradient induces shift of the expression domain of *krox20*. The expression of *krox20 *labels rhombomeres 3 and 5 (A, yellow). Co-injection of *lacZ *and *cyp26 *or *rdh10 *or *rdh2 *mRNA into one blastomere of two-cell stage *Xenopus *embryos is indicated by LacZ staining (B, C, blue). The uninjected side is served as control. Ectopic *cyp26 *at one side of the embryos causes posterior shift of *krox20 *(B, yellow). In contrast to *cyp26*, overexpression of *rdh10 *and *radlh2 *leads to anterior shift of *krox20 *(C, yellow). This diagram is based on the previous reports and our unpublished data [46,92,129] (RKT Kam, Y Chen, WY Chan and H Zhao. Dhrs3 attenuates the retinoic acid signaling and is required for early embryonic patterning. Submitted.).

Vertebrate hindbrain contains seven rhombomeres. Several *Hox *genes have been shown to be involved in rhombomeres formation and/or provide positional identity to specific rhombomeres [116]. Two RAREs have been identified in *Hoxb1*, one of which is located 5' to *Hoxb1 *promoter and is required for the restricted expression in rhombomere 4 [117]. Studies in mouse and fish embryos showed that retinoids could induce ectopic expression of *Hoxa1 *and *Hoxb1*, which caused a rhombomere 2 to 4 transformation [118-120]. In addition, the *Hnf1b *that suppressed *Hoxb1 *expression in rhombomere 5 was also regulated by the RA signaling [121,122]. Altogether, these data implicate that the RA signaling is involved in hindbrain patterning. Indeed, knockout mice lacking *Raldh2 *led to the conversion of caudal hindbrain segments into rhombomere 4 identity [123,124] and the same is true for RA antagonist treated mice [109]. In chick embryos, treatment by using increasing concentrations of RA antagonist, BMS453, showed that successively more posterior rhombomere boundaries required progressively higher concentration of endogenous retinoic acid for their correct positioning [125]. In zebrafish embryos, rhombomere 5 of the caudal hindbrain is well specified but both rhombomere 4 and 5 were posteriorly expanded in the absence of RA [42,126]. In *Xenopus *embryos, injection of *cyp26a1 *mRNAs into one side of the embryo resulted in alterations of the RA signaling at the injected side as indicated by posteriorization of *krox20*, a transcription factor marking the rhombomere 3 and 5 [92], whereas increasing the RA signaling via ectopic expression of *raldh2 *led to anteriorization of both midbrain and hindbrain rhombomere identities in the expense of some forebrain territory [38] (Figure 5). Despite this, studies in mouse and *Xenopus *showed that decreased RA signaling did not change the mid-/hindbrain boundary (MHB)[41,92], which requires the orchestration of a complex regulatory network involving the FGF, Wnt, and Shh signalings [127]. On the contrary, the hindbrain/spinal cord boundary seems to be specified depending on the RA signaling. In zebrafish *cyp26a1 *mutant embryos, the expression domains of *hoxb5a *and *hoxb6a *were expanded rostrally, leading to the expansion of rostral spinal cord at the expense of the hindbrain territory [128].

### 5.4 Pancreas

**Figure 6:**
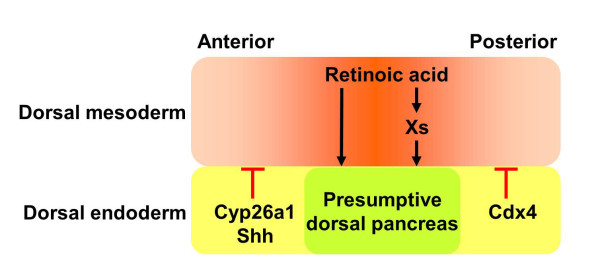
**RA plays an essential role in dorsal pancreas specification**. During gastrulation and early neurulation, the field in dorsal endoderm that gives rise to the presumptive dorsal pancreas is just underlying the dorsal mesoderm where the highest concentration of RA is generated. These RA signals can act to underly endoderm either directly, or indirectly via first activating unknown factors (Xs) in mesoderm, which then act on the endoderm for dorsal pancreas formation. As described in the text, *Shh *and *Cyp26a1 *expressed in more anterior dorsal endoderm block pancreas formation probably via inhibiting the RA signaling activity or directly degrading RA. *Cdx4 *expressed in the posterior dorsal endoderm helps to establish the posterior boundary of dorsal pancreas via antagonizing the RA signaling.

The RA signaling also plays essential roles in the regionalization of endoderm as early as during gastrulation [129]. Studies in frog, avian, and mice indicate that the RA signaling is essential for dorsal pancreas formation, partially required for ventral pancreas patterning [129-134]. In zebrafish, inhibition of RA signaling before the end of gastrulation inhibits the initial development of both hepatic and pancreatic endoderm [135]. Evidence shows that it is the RA signal emanating from paraxial mesoderm that directly induces endocrine pancreatic precursors in the adjacent endoderm [136]. For example, *Mnx1 *is identified as an RA downstream gene that controls cell fate choice in the developing endocrine pancreas [137]. *Cyp26a1*, expressed in endoderm, defines the anterior boundary of dorsal pancreatic anlage by inactivating excess RA [138], while *Cdx4*, expressed in posterior endoderm, acts to antagonize the RA signaling and thus establishes the posterior boundary of dorsal pancreatic territory in zebrafish embryos (Figure 6; [139]). Moreover, *Raldh1 *is expressed in developing mouse and human pancreas at stages when mature β-cells are generated [140], suggesting the role of RA in β-cell differentiation. The *XPDIp *is expressed in the exocrine portion of both dorsal and ventral pancreas. Inhibition of RA signaling by BMS453 treatment completely abolished expression *of XPDIp *in dorsal pancreas, but had mild effects on the ventral pancreas, suggesting the development of dorsal pancreas is more RA signaling dependent [129].

In addition, *in vitro *data have also demonstrated a role of RA in pancreas development. In a defined *in vitro *explant system, RA treatment of cultured mouse E10.5 dorsal pancreatic buds could induce the generation of *Ngn3+ *endocrine progenitor cells and stimulate their further differentiation into β-cells by activating a differentiation program that recapitulates the normal temporal program of β-cell differentiation [140]. In zebrafish, exogenous RA led to expansion of both exo- and endocrine cells, while in *Xenopus *embryos as well as cultured mouse embryonic pancreatic rudiments, exogenous RA led to an expansion of the endocrine cell population at the expense of the exocrine cells in the dorsal pancreas [129,141,142]. Similarly, the induction of pancreatic fate by RA in endodermalized naive ectoderm dissected from early *Xenopus *gastrulae only showed the generation of early insulin-expressing cells [143,144]. Therefore, cautions should be taken to apply RA at the right time and the right dose (close to physiological concentration in early embryos) for *in vitro *induction of mature pancreatic β-cells from embryonic stem cells.

### 5.5 Heart

The effect of RA on cardiogenesis was first demonstrated in quail that vitamin A deficiency could induce defective primitive heart development [145]. Further evidence shows that RA signaling is required for the formation of cardiac progenitors [146], and the correct modeling of the early heart field and the anteroposterior patterning of the heart is controlled by atRA through the action of *Isl1 *and *Fgf8 *signaling pathway [147,148]. Knockout mice embryos lacking *Raldh2 *showed an abnormal development of the second heart field (SHF) [149], which is a population of undifferentiated cardiac precursor cells originating from the pharyngeal mesoderm and lying medial to the cardiac crescent (the first heart field). The expression of SHF markers including *Isl1, Tbx1*, and *Fgf8*, was posteriorly expanded in these embryos [150], indicating that the RA signaling is required for the restriction of SHF. Defects in the formation of a proper heart tube might also partially explain why *Raldh2-/- *is embryonic lethal [148]. Studies in zebrafish indicated RA functions in the restriction of cardiac specification [149]. During this process, if RA signaling was inhibited in lateral mesoderm, the uncommitted cells could differentiate into myocardial progenitor cells instead of pharyngeal or pancreatic cells [149], leading to the expansion of cardiomyocytes.

In addition to the heart development, the RA signaling has also been implicated in heart regeneration in adult zebrafish. Gene profiling assay indicated that *raldh2 *was one of the most highly induced genes in the regenerating heart [151]. After the ventricular injury, *raldh2 *was activated in the endocardium, but its expression was subsequently restricted in endocardial cells at the injury site one day post-trauma as the repairing cardiogenesis began. By 7 days after amputation, the epicardial cells at the injury site also expressed *raldh2*. Inhibition of RARs or expression of an RA-degrading enzyme blocked regenerative cardiomyocyte proliferation [152]. These findings reveal essential roles of RA in repairing the damaged heart tissue and promoting cardiomyocyte proliferation.

### 5.6 Kidney

There is increasing evidence indicating that atRA plays important roles in kidney development [153-155]. The kidney field is derived from the intermediate mesoderm. The specification of renal progenitor cells is influenced by RA signals emanating from the paraxial mesoderm [153-155]. Recent studies in *Xenopus *and zebrafish have demonstrated that ectopic production of RA increased the size of the kidney field, while blocking the pathway inhibited the kidney specification [86,154]. In *Xenopus, pax8 *and *lhx1*,which are the earliest determinants of pronephric fate are both under control of the RA signaling [154,156], while *pteg*, an early pronephric marker, is a direct target of the RA signaling and an essential factor for pronephric specification [157]. Another critical factor for the pronephros development, Wilm's tumor suppressor (*wt1*), is under direct control of the RA signaling as well [158,159]. Inhibition of RA by BMS453 treatment caused reduction of pronephros field, as revealed by the decreased expression of the pronephros markers including *smp-30 *and *pax2 *at the late tailbud stage [154]. Furthermore, in *Xenopus *embryos, the pluripotent animal cap cells treated with RA and activin could differentiate into pronephros tissue [154,160]. Likewise, the RA signaling is also essential for directing the proximodistal patterning of pronephric nephron in zebrafish [161]. The exogenous RA could induce proximal segment fates at the expense of distal fates, whereas inhibition of the RA signaling caused a loss of the proximal segments and an expansion of the distal segments.

Interestingly, inactivation of both *RARα *and *RARβ *in mice embryos resulted in renal malformations [155]. Further evidence showed that *RARα *and *RARβ *were co-expressed with *Ret*, a receptor tyrosine kinase involved in renal development, in renal stromal mesenchyme, where their deletion led to altered stromal cell patterning, impaired ureteric bud growth, and down-regulation of *Ret *in the ureteric bud. Moreover, studies in mice indicate that the RA signaling in ureteric bud cells mainly depends on atRA generated through Raldh2 in stromal cells [162].

### 5.7 Lung

Evidence indicates that disruption of the RA signaling during early development caused abnormalities of lung formation. Either knockout of *Raldh2 *or treatment with BMS 493, a RA inhibitor, caused defects of lung formation in mice [163]. During primary lung bud morphogenesis, although RA is not required for specification of lung cell fate in the endoderm, it is essential for the induction of the primordial lung buds [163,164]. During the induction of lung bud, the RA signaling inhibits the expression of *Dkk-1*, the Wnt signaling inhibitor, in the foregut mesoderm. This inhibition in turn activates Wnt signaling in the lung bud region. At the same time, the RA signaling also inhibits TGF-β signaling. The balanced Wnt and TGF-β signalings coordinate with each other to induce and maintain the optimal expression level of *Fgf10 *for the induction of the lung primordium [165]. Furthermore, airway branching was accompanied by down-regulation of the RA signaling pathway [166], thus regional mechanisms that control RA availability and utilization are important for the lung morphogenesis.

## Concluding remarks

To date, it has been demonstrated that the RA signaling plays sophisticated roles in early embryonic development. In fact, gastrulation is a very critical period for the RA signaling to exert its function in the regionalization of all three germ layers along the anteroposterior axis. RA is mainly generated in the mesoderm and thus can be most efficiently used. It is not clear as to how the RA metabolic enzymes coordinate with each other to generate the RA gradient at right time, right region, and with right strength during early embryonic development. Future studies are needed to identify the immediate and direct RA target genes in distinct RA responsive cells and to define the crosstalks between RA and other signaling pathways, such as hedgehog, FGF, Wnt, and Notch signalings for the formation and regionalization of three germ layers. Such knowledge will be essential for understanding organogenesis and establishing reliable strategies for stem cell differentiation into specific cell types that can be used for treatment of human diseases.

## Competing interests

The authors declare that they have no competing interests.

## Authors' contributions

RKTK and HZ planned the manuscript outline. RKTK, HZ and YLC wrote the draft, YD revised and did proof reading, HZ and YLC finalized the manuscript. All authors read and approve the final manuscript.
